# Effect of repetitive transcranial magnetic stimulation on perioperative anxiety in female patients: study protocol for a single-center, randomized controlled study

**DOI:** 10.3389/fmed.2026.1832192

**Published:** 2026-04-23

**Authors:** Wenhui Wang, Sitong Zhou, Yunlai Wang, Yuxuan Huang, Huadong Ni, Ming Yao

**Affiliations:** 1 Department of Anesthesia Medicine, Zhejiang Chinese Medical University, Hangzhou, Zhejiang, China; 2 Department of Anesthesiology and Pain Research Center, The Affiliated Hospital of Jiaxing University, Jiaxing, China

**Keywords:** female patients, perioperative anxiety, protocol, randomized controlled study, repetitive transcranial magnetic stimulation

## Abstract

**Introduction:**

Perioperative anxiety is a widespread psychological issue among patients undergoing surgery and often has a significant impact on patient outcomes. Due to physiological and psychological characteristics such as hormonal fluctuations and higher emotional sensitivity, female patients generally have a higher incidence of perioperative anxiety compared to male patients. This investigation aims to examine the repetitive transcranial magnetic stimulation (rTMS) effects on perioperative anxiety in female patients.

**Methods:**

This single-center, randomized, regulated investigation will be performed at the Affiliated Hospital of Jiaxing University. The investigation plans to enroll 252 female individuals scheduled for elective surgery under general anesthesia, who will be classified in a random manner to active rTMS or sham rTMS. The primary outcome is the change in anxiety score from baseline to 3 days post-operation. Secondary outcome measures include anxiety scores, occurrence of postoperative delirium (POD), pain scores, frailty scores, and sleep quality scores. Intention-to-treat and per-protocol populations were employed to conduct analyses.

**Discussion:**

This research aims to estimate the influence of rTMS on perioperative anxiety in female patients. It is expected to offer a successful non-pharmacological treatment approach for clinical practice, alleviate anxiety symptoms in female patients during the perioperative period, and enhance the safety of surgical procedure and the quality of post-operative recovery for patients.

**Clinical trial registration:**

https://www.chictr.org.cn/index.html, identifier ChiCTR2500097991.

## 1 Introduction

Perioperative anxiety is a prevalent psychological issue among surgical patients, characterized by a diffuse sense of tension and unease stemming from factors such as the underlying disease, hospitalization, anesthesia, and uncertainty about the surgical procedure (1). It encompasses both preoperative and postoperative anxiety (2). The incidence of perioperative anxiety varies across different disease populations, ranging from 11 to 80% (3). Data from the National Institute of Mental Health indicate that females are more susceptible to various anxiety symptoms than males. Further research reveals that the occurrence of anxiety disorders in females is 1.3 to 2.4 times higher than in males, with this disparity being particularly pronounced during the perioperative period, where the incidence significantly exceeds that in males (4, 5). This gender difference involves both biological factors, such as hormonal influences, as well as psychological and sociocultural factors, including gender role expectations and stress coping strategies (6–8). In the surgical setting and under stressful conditions, the occurrence of perioperative anxiety is significantly increased in female patients, with anxiety levels often peaking on the night before or the morning of operation (5, 9). Preoperative anxiety increases both physiological and psychological burden, adversely affecting short- and long-term recovery outcomes. These include heightened postoperative pain, extended hospital stays, and delayed convalescence (10, 11). Moreover, anxiety is closely associated with other psychiatric conditions such as depression, cognitive decline, and sleep disturbances (12, 13). Thus, early intervention for pre-operative anxiety is important.

Pharmacological and non-pharmacological strategies are commonly employed in clinical practice to alleviate perioperative anxiety. Pharmacological interventions mainly include benzodiazepines, non-benzodiazepine anxiolytics, and antidepressants (1). Among benzodiazepines, midazolam is frequently the preferred option due to its short half-life and relatively favorable side effect profile. However, long-term use of benzodiazepines may lead to tolerance or dependence, and these agents have been associated with an increased risk of postoperative cognitive dysfunction and delirium (14). Non-pharmacological approaches encompass preoperative education, music therapy, and cognitive-behavioral techniques (CBT) (15). However, CBT typically requires multiple sessions and may not be feasible in the limited perioperative timeframe. Preoperative education and music therapy is safe and accessible but often yields modest effects (16). Non-invasive central neuromodulation technologies such as tDCS have also shown the potential to relieve perioperative anxiety, but the application of rTMS for perioperative anxiety remains limited (17, 18).

In 1985, Barker et al. (19) introduced transcranial magnetic stimulation (TMS), a non-invasive brain stimulation technique. By placing a coil on the scalp, TMS generates a magnetic field that penetrates the skull and induces electrical currents in the cerebral cortex, resulting in neuronal membrane depolarization, action potential generation, and modulation of cortical excitability in targeted brain regions (20). Repetitive TMS (rTMS), a specific form of TMS, delivers magnetic pulses at regular intervals. It is now widely used in the treatment of various neuropsychiatric disorders, and clinical research on its application in anxiety spectrum disorders continues to expand globally (21). Most studies have focused on its emotional and physiological effects on the dorsolateral prefrontal cortex (DLPFC) (22, 23). However, evidence about the rTMS effects on perioperative anxiety remains inadequate. Therefore, this investigation aims to estimate the impact of rTMS on perioperative anxiety in females and to provide clinical evidence supporting its use as an intervention for anxiety relief in the perioperative setting.

## 2 Methods and analysis

### 2.1 Study design

**Figure 1:**
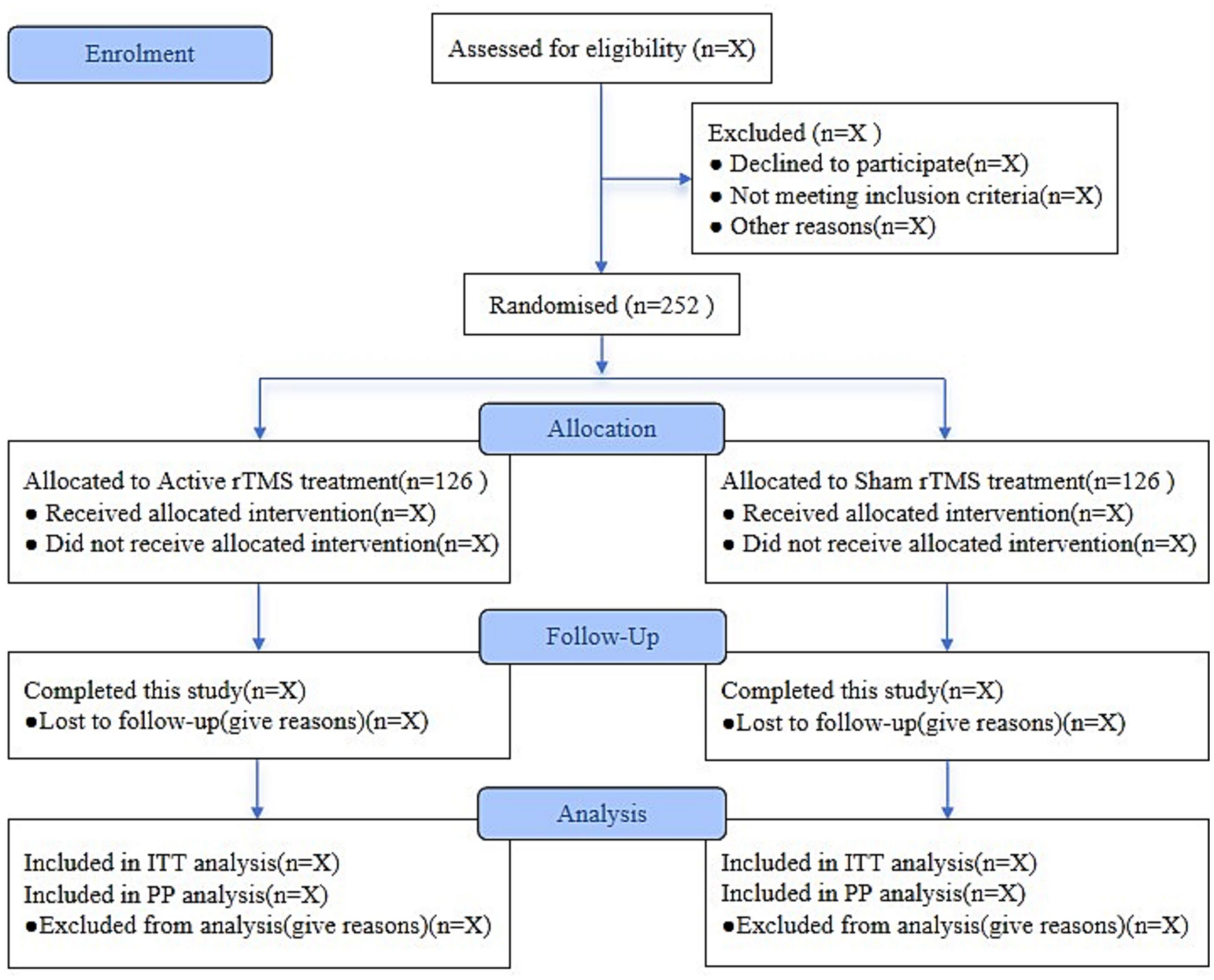
The flow chart of the study. rTMS, repetitive transcranial magnetic stimulation; ITT, intention-to-treat; PP, per-protocol.

This randomized controlled study will be conducted at The Affiliated Hospital of Jiaxing University. Participant recruitment is scheduled to begin on March 2, 2025. A total of 252 female patients undergoing elective surgery will be enrolled and randomly assigned in a 1:1 ratio to either the active rTMS group or the sham rTMS group. Ethical approval for the study was granted by the hospital’s Ethics Committee (Approval No. 2025-KY-049) on February 12, 2025. The trial was registered with the Chinese Clinical Trial Registry on February 14, 2025 (Registration No. ChiCTR2500097991). The schedule for enrollment, interventions, and assessments is presented in Table 1, and study design is illustrated in the flowchart (Figure 1).

**Table 1:** Study timeline.

Timepoint	Study period					
	On the day pre-operation	on the day of operation	T1	T2	T3	T4
Enrolment						
Inclusion criteria	**×**					
Exclusion criteria	**×**					
Informed consent	**×**					
Randomization	**×**					
Allocation						
Interventions						
Active rTMS group	**×**	**×**				
Sham rTMS group	**×**	**×**				
Outcome measurement						
HADS-A	**×**		**×**	**×**	**×**	**×**
NRS			**×**	**×**	**×**	
POD			**×**	**×**	**×**	
FRAIL			**×**	**×**	**×**	**×**
PSQI			**×**	**×**	**×**	**×**

PSQI, Pittsburgh Sleep Quality Index; NRS, Numeric Rating Scale; POD, postoperative delirium; rTMS, repetitive transcranial magnetic stimulation; FRAIL, Fatigue, Resistance, Ambulation, Illness and Loss of Weight Index; T1, the 1st day post-operation; HADS-A, Hospital Anxiety and Depression Scale-Anxiety subscale; T2, the 2nd day post-operation; T3, the 3rd day post-operation; T4, the 3rd month post-operation.

### 2.2 Eligibility criteria

#### 2.2.1 Inclusion criteria

1. Females ≥ 18 years old
2. Individuals scheduled for operation under general anesthesia
3. ASA grade ≤ III
4. Hospital Anxiety and Depression Scale-Anxiety (HADS-A) Scale score ≥ 8 points
5. Compliant with ethics, individuals voluntarily contribute to the trial and provide informed consent

#### 2.2.2 Exclusion criteria

1. Did not provide informed consent
2. Patient has a history of neuropsychiatric illness or previous neuropsychiatric disorder
3. History of craniocerebral or scalp injury or surgery
4. Have severe cardiovascular and cerebrovascular diseases
5. *In vivo* metal implants
6. Long-term use of psychotropic substances or abuse of alcohol or illicit drugs
7. Pregnant or lactating women

#### 2.2.3 Withdrawal or dropout criteria

Participants may leave from the investigation at any time for any reason with no penalty. Continuous care will be ensured, and reasons for withdrawal will be documented. The investigator will discontinue the participation of any subject if any of the following conditions occur during the study:

1. Failure to strictly follow the study plan
2. Failure to complete follow-up visits
3. Inability to tolerate repeated TMS intervention
4. Withdrawal from the study due to personal reasons

## 2.3 Randomization and blinding

This study utilizes a block randomization method. A computer-generated random block sequence of 4 or 6 will be used to centrally randomize participants into either the active rTMS group or the sham rTMS group at a 1:1 ratio. The randomization sequence was generated by an independent researcher from the study team using a computer-based program. This researcher had no involvement in participant recruitment, intervention delivery, outcome assessment, or data analysis. Sequentially numbered, opaque, sealed envelopes were used to ensure allocation concealment. After enrollment and baseline assessment, the envelope was opened by the operator responsible for administering the rTMS at the time of the first rTMS session to determine group assignment. To ensure effective blinding, both groups will use identical stimulation equipment and follow the same operating procedures. However, the device used in the sham rTMS group will not deliver pulsed magnetic fields to the brain; it will only produce sounds and vibrations similar to those generated during actual pulse stimulation. Throughout the study, all other parties, including the participants, outcome assessors, and data analysts, will remain blinded to group assignment.

## 2.4 Unblinding

Throughout the investigation, individuals will be closely observed throughout the investigation. Should any participant experience serious adverse reactions related to rTMS, such as dizziness, headache, nausea, vomiting, tinnitus, arrhythmia, or seizures, immediate unblinding will be performed to minimize potential bias.

## 2.5 Study interventions

All rTMS procedures were performed by professionally trained and experienced operators who had undergone standardized training prior to study initiation. The rTMS intervention is administered on the day before surgery and the day of surgery, resulting in two sessions per participant. This protocol is designed to accommodate the limited perioperative time window while providing a rapid, clinically feasible intervention.

Stimulation parameters are based on established neurophysiological principles. The right dorsolateral prefrontal cortex (DLPFC) is selected as the target region due to its role in negative emotional processing and its involvement in anxiety-related neural circuits. Low-frequency (1 Hz) stimulation is applied to reduce cortical excitability and modulate prefrontal–limbic pathways. The entire procedure strictly followed a unified protocol to ensure consistency in coil positioning, stimulation parameters, and patient interaction.

First, a circular coil was used to locate the stimulation target. The primary motor cortex (M1) was clinically identified as the area 2 cm anterior and 1–1.5 cm lateral to the intersection of the naso-occipital line and the temporoparietal line on the patient’s scalp (i.e., the Cz point). The resting motor threshold (RMT) for each patient was then determined as a reference for setting the stimulation intensity, expressed as a percentage of the rTMS device’s maximum output (24). The RMT was defined, using the visual observation technique, as the lowest stimulation intensity capable of eliciting a visible contraction in the target muscle in 5 out of 10 trials while the muscle was completely relaxed (24). Subsequently, a figure-8 coil was used to target the right DLPFC. The center of the coil was positioned parallel to the patient’s scalp, with its midpoint aligned directly over the stimulation site. The rTMS treatment was then delivered at an intensity of 80–120% of the RMT using a low frequency of 1 Hz (21). Both groups will undergo the intervention using identical devices and operational procedures. However, in the sham rTMS group, the device will not deliver pulsed magnetic fields to the brain but will only produce similar audible clicking sounds and scalp sensations/vibrations mimicking those emitted during active stimulation.

## 2.6 Anesthesia

Standard intraoperative monitoring is initiated upon patient arrival in the operating room [e.g., pulse oximetry, electrocardiogram (ECG), non-invasive blood pressure (NIBP), and bispectral index (BIS)].

General anesthesia is induced with 2.0–2.5 mg·kg^−1^ propofol, 0.3–0.5 μg·kg^−1^ sufentanil, and 0.6–1 mg·kg^−1^ rocuronium. Tracheal intubation is performed once BIS falls below 60, and mechanical ventilation is utilized to keep end-expiratory CO₂ between 35 and 45 mmHg. Anesthesia is continued with intravenous propofol (4–6 mg·kg^−1^·h^−1^), remifentanil (0.1–0.3 μg·kg^−1^·min^−1^), and 1% sevoflurane inhalation to keep BIS between 40 and 60. Vasoactive agents are administered as required to keep hemodynamic stability.

Postoperatively, patients are transferred to the post-anesthesia (PACU) or surgical intensive (SICU) care units, and extubation occurs upon recovery of consciousness. Analgesia is provided via patient-controlled intravenous analgesia (PCIA) using 100 μg sufentanil and 8 mg ondansetron diluted to 100 mL with normal saline, with a background infusion of 1.5–2.0 mL·h^−1^ and patient-controlled bolus doses of 1.0–2.0 mL.

## 2.7 Outcomes

### 2.7.1 Primary outcome

The primary outcome is the change in anxiety score from baseline to 3 days post-operation, measured using the HADS-A (25), with a score of ≥8 indicating clinically significant anxiety (26). The minimal clinically important difference (MCID) for the Hospital Anxiety and Depression Scale (HADS) is not fixed and varies across clinical contexts and analytical methods. Evidence from anchor-based studies and systematic reviews suggests that a change of approximately 1.5–2.0 points in the HADS total or subscale scores is generally considered clinically meaningful (27–29). Anxiety was assessed preoperatively in the ward on the day pre-operation (T0) prior to the first rTMS intervention, and the second intervention was administered in the pre-operative holding area on the day of operation. Postoperative anxiety scores were measured on days 1 (T1), 2 (T2), 3 (T3), and the 3rd month post-operation (T4).

### 2.7.2 Secondary outcomes

Secondary outcomes comprise: anxiety scores (T1–T4); occurrence of post-operative delirium [evaluated via the Confusion Assessment Technique for the ICU (30) during T1–T3]; pain scores [10-point Numeric Rating Scale (NRS) (31) during T1–T3, 0 = no pain, 10 = worst imaginable pain]; frailty scores [FRAIL Index (32) during T1–T4, 0 = most robust, 5 = most frail]; and sleep quality [Pittsburgh Sleep Quality Index (PSQI) (33) during T1–T4, 0–21, higher scores denote poorer sleep].

## 2.8 Adverse events and safety

All participants will undergo rigorous pre-enrollment screening to minimize potential risks associated with rTMS. Individuals with a history of epilepsy, major neurological disorders, or intracranial metal implants will be excluded. During each stimulation session, participants will be closely monitored for adverse symptoms (e.g., dizziness, headache, nausea, vomiting, tinnitus, arrhythmia, or seizures) (34). To ensure participant safety, an independent monitoring team was designated to oversee the study conduct, data quality, and adverse events. All adverse events are systematically recorded and reported to the principal investigator and the institutional ethics committee. The ethics committee retains the authority to recommend modification or termination of the study if necessary. Continuous safety monitoring is implemented throughout the study period, and predefined criteria for study discontinuation include the occurrence of serious adverse events or any unexpected safety concerns.

## 2.9 Data collection and management

After each participant visit, data will be recorded on paper Case Report Forms (CRFs) and subsequently entered into Microsoft Excel by an investigator not involved in the intervention. All study data will be securely stored in a separate, restricted-access location. The principal investigator will regularly review all adverse events and, when necessary, convene investigator meetings to evaluate the study’s risk–benefit profile. Unblinding procedures may be implemented if required to ensure participant safety and protect their legal rights. An independent data monitoring team will oversee the accumulated safety and efficacy data to determine whether the study should continue.

## 2.10 Sample size

We conducted a pilot study comparing active versus sham rTMS in female patients. The pilot results showed a mean difference in anxiety scores of 1.3 between groups, with a standard deviation (SD) of 2.9. Depending on a significance level of 0.05 and a power of 0.80, we calculated a sample size of 78 patients/group via PASS 15.0. Accounting for a 10% dropout rate and pre-specified subgroup analyses, we aimed to recruit 126 participants/group to confirm sufficient study reliability.

## 2.11 Statistical analysis

All analyses will be conducted according to the intention-to-treat (ITT) principle, with participants assessed in their originally assigned groups regardless of protocol adherence or treatment received. Baseline demographic and clinical characteristics will be summarized to evaluate balance between the groups. Continuous variables will be assessed for normality using the Shapiro–Wilk test. Normally distributed variables will be presented as mean ± standard deviation (SD) and compared using independent t-tests, while non-normally distributed variables will be expressed as median (interquartile range, IQR) and analyzed using the Mann–Whitney U test. Categorical variables will be expressed as counts and percentages and compared using the chi-square test or Fisher’s exact test.

The primary outcome will be analyzed using a linear mixed-effects model, including treatment group, time, and treatment-by-time interaction as fixed effects, and a random intercept for each subject to account for within-subject correlations. Baseline HADS-A score, age, type of surgery, and relevant perioperative variables (e.g., anesthesia duration) will be included as covariates to control for potential confounding.

Secondary outcomes will be assessed via a linear mixed-effects model, comprising baseline values as covariates. Fixed effects will involve treatment, time, and the treatment-by-time interaction, with a random intercept for subjects accounting for within-subject variability. The treatment-by-time interaction will be initially examined. If significant, group differences and within-group changes at each time point will be assessed, with Bonferroni correction applied for multiple comparisons of baseline changes in anxiety scores. If not significant, only the major effect of treatment will be evaluated, and no Bonferroni correction will be utilized for time-specific treatment effects. Binary outcomes will be compared via the *χ*^2^ test or Fisher’s exact test, with relative risk (RR) and 95% confidence intervals (CI) reported.

In the exploratory analyses, to investigate the heterogeneity of rTMS effects across subgroups within the anxious population, we performed a series of subgroup analyses for the primary outcome measure. The primary subgroup analysis was depending on the level of patient anxiety: individuals with a HADS-A score ≥8 were defined as having anxiety symptoms, with scores of 8–10 (mild), 11–14 (moderate), and 15–21 (severe). We also examined subgroup effects according to surgery type, PSQI score (≤15 vs. > 15), and FRAIL scale result (no frailty vs. pre-frailty or frailty). These analyses were conducted by adding an interaction term between the treatment group and the subgroup variable to the linear mixed-effects model for the primary outcome. Effect heterogeneity was examined by estimating the importance of interaction terms. The adjusted odds ratio (aOR) and its 95% CI for each subgroup were displayed in a forest plot.

## 2.12 Strategy for missing data

Throughout the clinical trial, any missing data will be carefully documented, with the reasons for and extent of missingness recorded. The pattern and mechanism of missing data will be evaluated to determine whether data are missing completely at random (MCAR), missing at random (MAR), or missing not at random (MNAR). If the loss to follow-up rate is below 10% during the follow-up period, the analysis will be based on complete-case data. If the loss to follow-up rate reaches 10% or higher, multiple imputation using chained equations (MICE) will be applied to account for potential bias under the MAR assumption.

## 3 Discussion

Perioperative anxiety, a common psychological complication associated with surgical procedures, has garnered increasing clinical attention due to its strong correlation with exacerbated postoperative pain, delayed recovery, extended hospital stays, and reduced patient satisfaction (15). Currently, benzodiazepines remain the primary pharmacological intervention; however, their use is associated with significant risks, including respiratory depression, excessive sedation, cognitive impairment, and potential for dependence (14). Therefore, there is a pressing clinical need to explore safe, effective, non-pharmacological alternatives that are also non-addictive.

Non-invasive brain stimulation techniques primarily include repetitive transcranial magnetic stimulation (rTMS), transcranial direct current stimulation (tDCS), and transcranial alternating current stimulation (tACS). Among these, rTMS is the most widely applied in clinical settings for various neuropsychiatric disorders. Its mechanism involves delivering a rapidly changing, high-intensity pulsed magnetic field to the scalp, which penetrates the skull and induces a localized electric field in the targeted cortical region (20). This, in turn, leads to neuronal depolarization or hyperpolarization, thereby modulating cortical excitability. Beyond its immediate effects, repeated rTMS sessions can induce synaptic plasticity changes akin to long-term potentiation (LTP) or depression (LTD), resulting in sustained neuromodulatory after-effects (20, 35). Clinical research on rTMS for treating anxiety spectrum disorders is increasingly growing both domestically and internationally in the field of psychiatry. Studies by Zwanzger et al. (36) has shown that anatomical structures such as the prefrontal cortex, temporal lobe, amygdala, and thalamus are involved in the generation of anxiety. Furthermore, functional brain research suggests a hemispheric specialization of the dorsolateral prefrontal cortex (DLPFC) in emotional processing: the left DLPFC is primarily implicated in the formation or control of positive emotions, whereas the right DLPFC is more involved in the generation or regulation of negative emotions (37). In patients with persistent anxiety, this balance is often disrupted, manifesting as hypoactivity in the left DLPFC and relative hyperactivity in the right DLPFC (38). The therapeutic effects of rTMS are highly parameter-dependent, with different stimulation frequencies producing opposing neuromodulatory outcomes. High-frequency rTMS (5–20 Hz) typically enhances cortical excitability, while low-frequency stimulation (1–5 Hz) tends to diminish it (39). Leveraging this frequency-dependent effect, rTMS offers a mechanistically targeted approach to directly intervene in the neural circuits underlying anxiety. Therefore, its application in the specific stressful context of the perioperative period represents a novel, non-pharmacological strategy for anxiety management, one that is grounded in a well-defined neurobiological rationale.

This study specifically focuses on the female patient population. Epidemiological evidence indicates that the prevalence of anxiety disorders is consistently higher in females than in males (5, 6). During the perioperative period, female patient’s exhibit heightened sensitivity to surgical stress due to a combination of hormonal fluctuations and psychosocial factors, resulting in more pronounced anxiety compared to their male counterparts (8, 17). Therefore, concentrating the study population on females helps control for gender as a potential confounding variable, enhances the homogeneity of the research findings, and provides more targeted evidence for the future development of precision perioperative anxiety management protocols specifically for female patients. To ensure the reproducibility and scientific rigor of the intervention, this protocol provides a clear definition of the rTMS parameters. Based on established rTMS treatment guidelines and relevant clinical studies, low-frequency (1 Hz) stimulation targeting the right dorsolateral prefrontal cortex (DLPFC) was selected as the intervention protocol (40, 41). This stimulation paradigm has been shown to effectively modulate DLPFC excitability, thereby downregulating hyperactivity in subcortical regions such as the amygdala via the prefrontal-limbic pathway, ultimately alleviating anxiety symptoms (39, 42). Accordingly, this single-center, prospective, randomized, sham-controlled study aims to evaluate the effect of rTMS on perioperative anxiety in female patients.

Compared with previous studies on rTMS for anxiety, most existing research has been conducted in psychiatric settings, involving longer treatment durations and higher cumulative stimulation doses. In contrast, our study focuses on the perioperative population and adopts a brief, two-session protocol, which significantly reduces the treatment burden and is more suitable for the time-sensitive perioperative setting. Compared with conventional pharmacological approaches, rTMS offers distinct advantages, as it avoids risks such as respiratory depression, drug interactions, and postoperative cognitive impairment. Although this field is still in an exploratory phase and further large-scale randomized controlled trials are needed to optimize stimulation protocols and verify the durability of its therapeutic effects, preliminary clinical evidence suggests that rTMS holds promise as an alternative or adjunctive strategy for improving perioperative anxiety.

This study has several inherent limitations. First, as a single-center trial, the findings may be influenced by local patient characteristics, clinical practices, and healthcare system factors, thereby limiting external validity, therefore, multicenter studies are warranted to validate the broader applicability of the findings. Second, the follow-up period was limited to 3 months, which primarily captures short-term outcomes. Given that rTMS may exert cumulative effects through neuroplastic mechanisms, longer-term follow-up is required to evaluate sustained anxiolytic benefits and potential prevention of chronic anxiety or post-traumatic stress. Third, this study relied primarily on subjective assessment scales without incorporating objective biomarkers. The absence of neuroimaging or electrophysiological measures limits our ability to elucidate the underlying neural mechanisms of rTMS. Integrating rTMS with such measures in future research could provide deeper insights into its effects on brain function and facilitate the development of personalized perioperative anxiety management strategies. Fourth, although a sham-controlled design was employed, expectation bias cannot be entirely excluded. Sensory experiences associated with rTMS may influence patient-reported outcomes. Finally, excluding patients with neuropsychiatric comorbidities enhances internal validity but limits clinical applicability, given that such comorbidities are common in surgical populations. Therefore, future studies should include more heterogeneous cohorts to improve generalizability.
